# Neurology

**Published:** 1901-12

**Authors:** William C. Krauss

**Affiliations:** Buffalo, N.Y.


### Neurology.

Conducted by WILLIAM C. KRAUSS, M. D., Buffalo, N. Y.

### INSANITY AS A DEFENSE IN CRIMINAL CASES

Eastman, B. D., Topeka, Kas. (*Medical Age, October 10, 1901*), says: There are five points of view from which insanity as a defense in criminal cases will be considered: (1) the judicial; (2) the medical; (3) the popular; (4) the individual; (5) the expert.

*The Judicial View.*—Knowledge of right and wrong is the judicial test as to responsibility for crime. Every person by the common law is supposed to be sane and responsible unless the contrary is shown by the party pleading insanity as a defense. To make it a successful defense, insanity must be shown to be the result of disease either congenital or acquired, and to be of such extent as to destroy the power of distinguishing between right and wrong as to the particular act in question. It is not sufficient to claim that the accused has been impelled to commit crime, but he must be unable to discover or appreciate his legal and moral duty regarding the act in question. Indeed, as to moral influences impelling to crime, the bench has laid down the dictum: "Every crime was committed under an influence of such a description; and the object of the law was to compel people to control these influences." (Rolf. B. in Rogers vs. Hemet.) Judge Cox in his charge to the jury in the Guiteau case said:

> An irresponsible insane man can no more commit murder than a sane man can do so without killing. His condition of mind cannot be separated from the act. If he is laboring under disease of the mental faculties, if this is a proper expression, to such an extent that he does not know what he is doing, or does not know that it is wrong, then he is wanting in that sound memory and discretion which make part of the definition of murder.

The judge further states that if insanity be established to the degree which may be called total insanity, the defense is perfect. But it is in the so-called cases of partial insanity—for want of a better term—that difficulty arises. For a person afflicted with so-called partial insanity may commit offenses with which his infirmity has nothing to do; or he may commit a criminal act the nature and consequences of which he fully understands, and to the doing of which he is led by the same considerations that ordinarily govern in such cases.

Time will not allow an extended reference to judicial utterances on this question, and while the dictum laid down in the celebrated McNaughton case, that knowledge of right and wrong determines responsibility, has been somewhat mitigated in recent years, it still constitutes the substantial basis of judicial utterance.

*The Medical View.*—Many medical writers held to the view that whenever there is any mental disease the party is entirely irresponsible, and insist that there is no distinction between medical insanity and "legal insanity." On this subject Spitzka says:

On some occasions the question of defining what is called 'legal insanity' may be presented to the reader of these lines. When that question is asked, he may safely challenge the questioner to show him a broken leg or a case of smallpox in a hospital ward, which is not a broken leg or a case of smallpox in law; to show him a tumor or a softening of the brain, which is meningitis or sclerosis in law, or to define the conditions under which any disease-symptom becomes an indication of health. When these conditions are complied with, and not till then, may the physician attempt to define 'insanity in law' as distinguished from insanity in science. In the meantime, he may rest contented with the dictum of one of the best legal authorities that that cannot be sanity in law which is insanity in science, just as nothing can be a fact in science, and a fiction in law at one and the same time.

But there is a growing tendency among psychiatrists to look upon this view as too extreme to meet the practical every-day requirements. Theoretically, every person who is mentally diseased in any degree is of unsound mind, just as every one who is affected with any physical infirmity is of unsound body. But a person may fall short of perfect soundness of body and not be incapacitated for general physical activity. It may be only when his infirmity is brought in contact with a certain class or standard of material demands, that his physical impairment becomes manifest and he exhibits physical incapacity. A broken leg is a broken leg both in medicine and law, but a medically diseased leg need not be a legally useless one.

There is no question that in case of total insanity (as expressed by Judge Cox) there is total irresponsibility. It is in those cases which lie along the border-line between sanity and insanity, and when at times the mind may be controlled by ordinary considerations, and at others be under the sway of unusual, morbid, irrational impulses, that doubt and difficulty arise. Just as bodily disease may exist to an extent which requires medical treatment but does not incapacitate for most of the ordinary physical responsibilities, so there may be a limited mental impairment which demands medical treatment, but does not ordinarily incapacitate for most of the moral responsibilities. It is only when some unusual psychic demand is made that mental incapacity is clearly manifested. The border-land of insanity, the debatable ground between complete responsibility and clearly admitted irresponsibility, is a broad step with ill-defined, irregular, ever-shifting boundaries.

*The Popular View.*—The popular view is variable, volatile and impulsive. It is as likely to err one way as the other. In a well-marked case of total and previously recognised insanity the popular view will generally be correct, but in other cases the public may jump at conclusions that will be erroneous. In the one case the popular verdict may hold the perpetrator sane and responsible because insanity has not been publicly manifested, when in fact it undoubtedly exists; in another case atrocity is held to show insanity when every indication points to deliberate, predetermined, responsible action. Such conclusions depend often upon the confounding of the unnatural condition which generally determines criminal acts with an irresponsible one due to disease. Thus, in a case of alleged patricide, the atrocious circumstances led many to say that if the party charged therewith committed the act he must have been insane, when the circumstances were such that an admission that the act was committed as charged necessarily carried with it an admission that the party accused thoroughly comprehended the nature and consequences to himself by aid of an intermediary.

Another phase of the popular view which not unfrequently sways a jury in deciding upon a verdict is in reality simply a sentimenal justification of the act in question, as when a man wreaks his vengeance on the despoiler of his home or seducer of his daughter.

*The Individual View.*—When a person has been arraigned for the commission of a serious crime—homicide, for instance—for which he has no other defense, his friends, his lawyer, and frequently he himself turn to insanity. It is all at once brought out that he has been subject to "spells," and whether these "spells" be actual attacks of insanity or epilepsy, or are due to drink, to violent, unrestrained temper, or to inherent viciousness, they are made to do duty as indicative of irresponsibility. In this way much discredit is brought upon the legitimate use of insanity as a defense. The rule is that the really insane do not admit their insanity.

*The Expert View.*—These different views of insanity come more or less in conflict whenever insanity is alleged as a defense in any criminal trial. The end and aim of judicial procedure is, or should be, to arrive at the truth. One of the methods by which it is sought in criminal trials is to enlighten the court; counsel often attempt to use the expert to confound the jury.

The great difficulty with which the expert witness has to contend, and which often misleads and confuses the jurors or induces them to ignore his testimony, is that he is selected or "called" as it is termed, in the interest of one side. He becomes, often unconsciously, amalgamated to the side which calls him, and being obliged to give his testimony in answer to a shrewdly formulated hypothetical question, he is prevented from giving the jury and court the assistance which it should be his proper function to render.

Various methods have been suggested as likely to enhance the real value of expert testimony and still preserve the rights of each party. In my opinion, the best way to secure competent, unbiased, helpful expert testimony, would be a provision of law authorizing the judge in every case where insanity is alleged as a defense to appoint one or more experts to thoroughly examine the case and report their finding to the court. Such experts would, like the judge, be impartial, would seek to ascertain the truth, and, like the judge, should be paid by the State a reasonable compensation irrespective of their finding. Such finding would be what all expert testimony ought to be, a genuine expert aid to the court.

The finding should contain the facts upon which it is founded and explain the meaning of such facts, and counsel should be allowed to introduce testimony as to the correctness of these alleged facts and their bearing upon the case. The experts should be allowed to fully explain their opinions, but the distorted hypothetical question and the badgering, often ridiculous, cross-examination should not be allowed. It may be claimed that in order to accomplish these results changes in our laws would be requisite. Then let us have the changes.

---

### INFECTIOUS DISEASES AND EPILEPSY.

TRURNOWSKY, M., reports in the *Wiener Med. Wochenschrift*, 35, 1901, three interesting cases in which infectious disease apparently cured the patient of epilepsy. The first patient was 38 years of age, and had suffered from epilepsy since her sixteenth year. In her twenty-third and twenty-fourth years she often had as many as fifteen to twenty seizures in a day. These seizures were typically epileptic, and so severe that two or three days were often required for complete recovery from one of them. After a very severe and long-continued seizure, on one occasion, the patient was attacked with a severe chill, followed by the development of a right sided croupous pneumonia. She made a normal recovery, and in the fourteen years that have since elapsed she has not had a single epileptic attack. Her functions are all normal.

The second case was a woman 34 years old, who had suffered from chorea in her eighth year, so severe that she could not eat without assistance. In her seventeenth year the St. Vitus dance ceased. In her eighteenth year she married. After this she aborted in the eighth month of pregnancy. One month later she had her first attack of epilepsy. She had two or three, and later five or six seizures a month. In 1891 she had a right-sided pneumonia, and from that time to the present she has been free from epilepsy.

The third case was a boy of 12 years, who, since the middle of his second year, had suffered from ten to fifteen severe epileptic seizures daily. His mental development had been greatly retarded; he was, in fact, an idiot, making only inarticulate sounds and devoid of habits of cleanliness. In his sixth year he had scarlet fever, and in the six years since that time he has not had an epileptic seizure. He has learned to speak fairly well, has improved intellectually and is in excellent health.

From the results in these cases the author concludes that it would not be reprehensible to place an epileptic in a position favoring the contraction of pneumonia, or to place an epileptic child in the same bed with one suffering from scarlet fever during a mild epidemic of the disease.—*Lancet-Clinic.*

---

### A CASE OF DELAYED APOPLEXY FOLLOWING TRAUMA.

BRUNS, O., (*Deut. Med. Woch.*, Sept. 12, 1901,) reports a case of a man 41 years old, who was struck on the head while at work. He did not become unconscious, but went on with his work, complaining to his fellow workmen of some headache. Four days afterward he was seized with a spell of sneezing, after which vertigo, vomiting and complete unconsciousness set in. He died the next day. The autopsy revealed a marked cerebral hemorrhage in the right hemisphere due to the rupture of a small aneurism near the corpus striatum. The author says that death was either due to the rupture of the vessel simply without any relationship to the blow, or that the trauma produced the acute aneurism and the sneezing caused its rupture. He believes the latter to be the cause which makes the sixth reported in the literature.—*Med. Age.*

